# Biogenic selenium nanoparticles and selenium/chitosan-Nanoconjugate biosynthesized by *Streptomyces parvulus* MAR4 with antimicrobial and anticancer potential

**DOI:** 10.1186/s12866-023-03171-7

**Published:** 2024-01-12

**Authors:** Mervat G. Hassan, Mariam T. Hawwa, Dina M. Baraka, Hamed M. El-Shora, Ahmed A. Hamed

**Affiliations:** 1 Botany and Microbiology Department, Faculty of Science, Benha University, P. O. Box 13511, Banha, Qalyubia Egypt; 2 Botany Department, Faculty of Science, Mansoura University, P. O. Box 35516, Mansoura, Dakahliaو Egypt; 3 Microbial Chemistry Department, National Research Centre, 33 El-Buhouth Street, P. O. Box 12622, Giza, Dokki Egypt

**Keywords:** *Streptomyces parvulus*, Biosynthesized selenium nanoparticles, Selenium/chitosan-nanoconjugate, Antimicrobial, Enzymes, Anticancer

## Abstract

**Background:**

As antibiotics and chemotherapeutics are no longer as efficient as they once were, multidrug resistant (MDR) pathogens and cancer are presently considered as two of the most dangerous threats to human life. In this study, Selenium nanoparticles (SeNPs) biosynthesized by *Streptomyces parvulus* MAR4, nano-chitosan (NCh), and their nanoconjugate (Se/Ch-nanoconjugate) were suggested to be efficacious antimicrobial and anticancer agents.

**Results:**

SeNPs biosynthesized by *Streptomyces parvulus* MAR4 and NCh were successfully achieved and conjugated. The biosynthesized SeNPs were spherical with a mean diameter of 94.2 nm and high stability. Yet, Se/Ch-nanoconjugate was semispherical with a 74.9 nm mean diameter and much higher stability. The SeNPs, NCh, and Se/Ch-nanoconjugate showed significant antimicrobial activity against various microbial pathogens with strong inhibitory effect on their tested metabolic key enzymes [phosphoglucose isomerase (PGI), pyruvate dehydrogenase (PDH), glucose-6-phosphate dehydrogenase (G6PDH) and nitrate reductase (NR)]; Se/Ch-nanoconjugate was the most powerful agent. Furthermore, SeNPs revealed strong cytotoxicity against HepG2 (IC_50_ = 13.04 μg/ml) and moderate toxicity against Caki-1 (HTB-46) tumor cell lines (IC_50_ = 21.35 μg/ml) but low cytotoxicity against WI-38 normal cell line (IC_50_ = 85.69 μg/ml). Nevertheless, Se/Ch-nanoconjugate displayed substantial cytotoxicity against HepG2 and Caki-1 (HTB-46) with IC_50_ values of 11.82 and 7.83 μg/ml, respectively. Consequently, Se/Ch-nanoconjugate may be more easily absorbed by both tumor cell lines. However, it exhibited very low cytotoxicity on WI-38 with IC_50_ of 153.3 μg/ml. Therefore, Se/Ch-nanoconjugate presented the most anticancer activity.

**Conclusion:**

The biosynthesized SeNPs and Se/Ch-nanoconjugate are convincingly recommended to be used in biomedical applications as versatile and potent antimicrobial and anticancer agents ensuring notable levels of biosafety, environmental compatibility, and efficacy.

## Introduction

Over the past few decades, significant concerns regarding cancer and resistant bacteria in the global healthcare system have become more challenging [1, 2]. As a result of misusing antibiotics, antimicrobial resistance (AMR) has been growing, leading society into the “post-antibiotic era” [3]. Furthermore, current studies indicated that tumors could become resistant to chemotherapy, much like bacterial resistance against traditional pharmaceuticals [4]. On the other hand, the current cancer treatments have reported numerous side effects on patients’ health [5]. Therefore, we are in desperate need of an immediate remedy different from the outdated and conventional ones.

Currently, nanotechnology is employed in various human-related sectors, such as biomedical, nutritional, chemical, biological, mechanical, optical, environmental, and agricultural [6–8]. Due to their outstanding functionality and reactivity, metal nanoparticles (NPs) have been widely used in various biomedical purposes, including antibacterial, antioxidant, anticancer, anticoagulant, or carriers for bioactive compounds [8–11].

Several methods have been reported for NPs synthesis, including chemical, physical, and biological [12]. The chemical and physical processes are usually complicated and expensive causing the release of hazardous byproducts that threaten ecological systems [13]. In contrast, biological method using biogenic agents, such as biopolymers, plant extracts, microorganisms, algae, or their derivative could successfully overcome most problems with chemical and physical methods, providing simple, environmentally friendly, high yielded, and economical approaches [14–16]. Actinomycetes are Gram-positive bacteria with high polydispersity, strong stability, and easy handling [17]. Actinomycetes, in particular *Streptomyces* sp., produce several secondary metabolites, such as enzymes and proteins, that can be employed for ion reduction and capping of metals at nanoscale [18–21].

Selenium (Se) is a crucial micronutrient which maintains human health and body functions (with a small range between toxic levels and nutritional deficiency of 400 and 40 μg/day, respectively) [22]. Additionally, Se has exhibited potent antioxidant, antibacterial, and anticancer properties [23, 24]. Compared to either inorganic or organic Se, selenium nanoparticles (SeNPs) are more biocompatible and less toxic [25–29]. SeNPs have been demonstrated to be a prospective therapeutic agent with drug delivery, anticancer, antimicrobial, antioxidant, anti-inflammatory, catalytic, and photoreactive properties [30–33].

Chitosan (Ch) is a nontoxic polycationic biopolymer which has a wide range of biological uses because of its unique chemical origin, positive charge, and presence of reactive hydroxyl and amino groups. Ch can be obtained by deacetylation of chitin which is found in shells of crustaceans (crab, lobster, and shrimp) and various organisms (insects and fungi). Furthermore, Ch is marketed for its exceptional properties, including biodegradability, biocompatibility, ability to form films, adsorption, wound healing, antibacterial, anticancer, and antioxidant [34–36].

Nano-chitosan (NCh) combines the advantages of NPs with the properties of Ch [37, 38]. Due to its remarkable biochemical properties (e.g., minimum toxicity, biocompatibility, biodegradability, antimicrobial/anticancer activity, and synergism with bioactive molecules), NCh has drawn much interest [39–42]. NCh has the advantage of drug release control, which boosts therapeutic efficacy and increases drug solubility and stability. Additionally, integrating nanometals into biopolymer matrix can enhance their biocompatibility and safety. Therefore, NCh can be used as a drug carrier with numerous prospects [43–45].

Microbial growth is significantly controlled by a set of metabolic key enzymes, such as phosphoglucose isomerase, pyruvate dehydrogenase, glucose-6-phosphate dehydrogenase, and nitrate reductase [46]. Phosphoglucose isomerase (PGI, EC 5.3.1.9) is an enzyme that converts glucose-6-phosphate (G6P) into fructose-6-phosphate (F6P), crucial for various cellular processes, including glycolysis and pentose phosphate pathway [47]. Pyruvate dehydrogenase complex (PDH) is a group of three enzymes that work together to convert pyruvate into acetyl-CoA involved in cellular respiration [48]. Glucose-6-phosphate dehydrogenase (G6PD) represents a cytosolic enzyme in pentose phosphate pathway that provides cells (like erythrocytes) by reducing energy [49]. Nitrate reductase (NR) catalyzes the nitrate conversion into nitrite while in bacteria and fungi, nitrite reductase converts nitrite to ammonium using NAD(P)H as an electron donor [50]. Consequently, inhibiting these enzymes could be considered as one of antimicrobials mechanisms.

Herein, the purpose of this study was to synthesize NCh, biosynthesize SeNPs by marine actinomycetes, conjugate these bioactive agents, and evaluate their antimicrobial activity with determining their inhibitory effects on microbial metabolic key enzymes as well as investigating their anticancer activity against different tumor cell lines.

## Material and methods

### Material

Starch nitrate agar, lysogeny broth, and ISP2 media were purchased from HiMedia laboratories, Mumbai, India. Tripolyphosphate (TPP), chitosan, sodium selenate (Na_2_SeO_4_), RPMI-1640 media, DMSO, nalidixic acid, nystatin, and cell lines were acquired from Sigma-Aldrich Co. (St. Louis, MO, USA). Fetal bovine serum (FBS) and MTT were obtained from GIBCO, UK. The Qiagen DNeasy Blood & Tissue Kit were gotten from Qiagen, Hilden, Germany. All solvents, buffers and reagents were obtained from El Nasr Pharmaceutical Chemicals Company (Cairo, Egypt).

### Marine samples collection and actinomycetes isolation

Samples were collected from marine sediments in Hurghada, Egypt. The collected samples were coded and photographed before being transported in a cooled sterilized container to the microbiology lab at the National Research Center and maintained at 4 °C. Marine actinomycetes were isolated using starch nitrate agar medium with 50% salt water and following components (g/L): 20 starch, 1 KNO_3_, 0.5 K_2_HPO_4_, 0.01 FeSO_4_, 0.5 MgSO_4_ 7H_2_O, and 15 agar [51, 52]. The medium pH was adjusted to 7 before sterilization. To inhibit the growth of bacteria and fungi, 20 μg/ml nalidixic acid and 50 μg/ml nystatin were then added. On the other hand, about 1 g of marine sediments were mixed with 10 ml of 0.8% sterile saline solution, and the mixture was agitated in an orbital shaker (100 rpm) for 30 min at 30 °C. The shaken suspensions were serially diluted (10^−1^ to 10^−6^), after which 0.1 ml of an appropriate dilution was spread on a medium plate with a sterile glass rod. The inoculated plates were incubated for 7 days at 30 °C. After incubation, actinomycetes colonies were selected and purified by streaking method. For later usage, the purified strains were stored at 4 °C on starch nitrate slants.

### Biosynthesis of selenium nanoparticles (SeNPs)

Based on their growth and morphology, eight actinomycetes strains were chosen to be screened for their ability to synthesize SeNPs extracellularly [53]. They were inoculated in 250 ml flasks with 50 ml of ISP2 media with the following ingredients (g/L): 4.0 dextrose, 4.0 yeast extract, and 10.0 malt extract at pH 7.2. The inoculated flasks were incubated for 7 days at 30–32 °C in a rotary shaker (200 rpm). To separate the supernatants, the cultures were centrifuged at 12,000 rpm for 15 min. Thereafter, 50 ml of 4 mM sodium selenate (Na_2_SeO_4_) aqueous solution (Sigma Aldrich, USA) was mixed with each supernatant (50 ml), and the reaction mixtures were incubated at 37 °C for 48 h. The visual evidence of SeNPs production was provided by the color change of the solution from pale yellow to deep orange [54, 55]. Consequently, the deep orange colloidal supernatant of a potential strain coded as LG (supernatant (LG)) was utilized for subsequent tests. A portion of this solution was centrifuged for 30 min at 8000 rpm to separate SeNPs and then dried for later use.

### Identification of potential strain

The LG actinomycete isolate was identified morphologically using transmission electron microscopy (TEM, JEOL GEM-1010) after 14 days of incubation on starch-nitrate agar medium. Additionally, for confirmation, the LG isolate was genetically identified. The DNA was purified after being extracted by the Qiagen DNeasy Blood & Tissue Kit in the same manner as the manufacturer’s recommendations. Using the primers 27F (5′-AGTTTGATCCTG GCTCAG-3′) and 1492R (5′-ACGGCTACCTTGTTACGACTT-3′), the 16S rRNA gene was annealed before being amplified by taq polymerase. The following PCR reaction conditions were used: 94 °C for 45 s, 55 °C for 60 s, and 72 °C for 60 s. The purified PCR products were sequenced at the Macrogen company in south Corea. In order to calculate the similarity score, the multiple alignments were aligned with other identified strains in GenBank database through the online BLAST program (http://www.blast.ncbi.nlm.ni.h.gov/Blas). Following that, the phylogenetic tree was constructed using the MEGA-X software [56].

### Preparation of nano-chitosan (NCh) and nanoconjugate

NCh was synthesized using ionic gelation with tripolyphosphate (TPP) crosslinking as described previously [57]. The TPP solution (0.5%, w/v in DW) was gently added by syringe needle (at 0.3 ml/min rate) while the Ch (0.4%, w/v) dissolved in acetic acid (0.5%, v/v) solution was being vigorously agitated. This process continued until the ratio of the chitosan: TPP solution reached 3:1, respectively. After TPP dropping, the stirring was maintained for 75 min. The produced NCh was then collected using speed centrifugation (10,500 rpm) for 30 min and rewashed with DW. For nanoconjugate formation by the biosynthesized SeNPs and NCh (hereafter mentioned as Se/Ch-nanoconjugate), produced powder of NCh (0.1%, w/v) was dissolved in 1% acetic acid solution. Then, 10 ml of the previous NCh solution was mixed with 50 ml of SeNPs (40 μg/ml), and the mixture was agitated for 90 min. After that, the resulting Se/Ch-nanoconjugate was precipitated by centrifugation, washing with DW, re-centrifugation, and ultimately freeze-drying.

### Characterization of synthesized nanoparticles (NPs) and nanoconjugate

The biosynthesis of SeNPs was confirmed by a clear peak seen in the solution absorption spectrum compared to the supernatant (LG) spectrum using UV-visible spectroscopy (JASCO V630 spectrophotometer) [58]. The crystal structure of SeNPs were investigated by X-ray diffraction (XRD) using a PAN analytical X’pert PRO X-ray diffractometer (Philips, Eindhoven, Netherlands), operating at 40 kV and 30 mA with Cu Ka1 radiation. The 2θ scanning range was 10° to 80° at 0.02°/min [59]. For functional characterization of SeNPs, NCh, Se/Ch-nanoconjugate, and supernatant (LG), Fourier transform infrared spectroscopy (FTIR) was conducted using FTIR 6100 spectrometer (Jasco, Japan). The tested NPs were ground with KBr. Then, the infrared spectra were recorded in 4000–400 cm^−1^ wavenumber region using a Broker vertex 80 v with a resolution of approximately 4–8 cm^−1^ [60]. Their structural features were determined using TEM imaging at 80 kV at the Regional Center for Mycology and Biotechnology (RCMB) in Al- Azhar University. For preparation, a drop of sample solution was put on carbon-coated copper grids (CCG) and allowed to drain slowly at room temperature before the TEM micrograph was taken [61]. Additionally, their morphology was further demonstrated using scanning electron microscopy (SEM) (Quanta FEG-250, Netherlands); the samples were prepared in a purified and dried powder before investigation. Furthermore, energy dispersive X-ray (EDX) analysis was performed to investigate their elemental composition at a 10 kV acceleration voltage [54]. Using Dynamic Light Scattering (DLS) technique [62], the hydrodynamic diameter, polydispersity index (PDI), and surface zeta (ζ) potential for biosynthesized SeNPs and Se/Ch-nanoconjugate were measured by Zetasizer (Malvern Nano ZS instrument, Southborough, MA).

### Antimicrobial activity

Antimicrobial activity of SeNPs, NCh, Se/Ch-nanoconjugate was assessed in 96-well flat polystyrene plate using wide range of microbial pathogens, including Gram-negative bacteria (*Salmonella typhi* ATCC-9992, *Proteus vulgaris* ATTC7829, and *Escherichia coli* ATCC25955), Gram-positive bacteria (*Staphylococcus aureus* NRRL B-767), fungi (*Aspergillus flavus* NRRLA326*, Aspergillus niger* AN512*, and Rhizoctonia* sp. Cy064), and yeast (*Candida albicans* ATCC10231) [52, 63]. To 180 μl of lysogeny broth (LB), 10 μl of tested NPs was added first, followed by 10 μl of log-phased microbial suspension. Then, the inoculated plate was incubated for an overnight period at 37 °C. After incubation, the clearance in the wells revealed tested NPs that had high antimicrobial activity, whereas the opacity in the wells determined NPs that had low antimicrobial activity. The Spectrostar Nano Microplate Reader (BMG LABTECH GmbH, Allmendgrun, Germany) was later employed to measure the absorbance.

## Influence of synthesized NPs on metabolic enzymes activity of tested microbial pathogens

### Preparation of enzymes extracts

According to El-Shora et al. [64], fungal enzyme extracts from *A. flavus*, *A. niger*, and *Rhizoctonia* sp. were prepared. Whatman filter paper No. 1 (Whatman, Piscataway, NJ, USA) was used to filter fungal mycelium, which was then washed by distilled water. About 10 g of mycelium was immersed in a 50 mM phosphate buffer (pH 7.0) with 5 mM cysteine for 30 min before being centrifuged at 5000 rpm for 10 min at 4 °C. For later use, the supernatant was utilized as a crude enzyme and kept at − 20 °C. The preparation of *C. albicans* extract was performed as described by Lima et al. [65]. About 300 ml of 200 mM NaHCO_3_ was added to 100 g of lyophilized yeast, and the mixture was agitated in an orbital shaker (300 rpm) at 35 °C for 24 h. The supernatant was then separated by centrifugation at 3000 rpm for 15 min.

*S. aureus*, *E. coli*, *S. typhi*, and *P. vulgaris* were inoculated in LB media and incubated overnight at 30 °C. After incubation, the cultures were centrifuged at 8000 rpm, and the collected bacteria were rinsed once with 50 mM phosphate buffer (pH 7.0). The bacterial cells were resuspended in 15 ml of buffer before being disrupted using discontinuous ultrasonication (every 10 s of ultrasonication at an intensity of 40% followed by a 20 s break) in an ice bath for 40 min. The supernatants, obtained from centrifugation at 10,000 rpm for 30 min, were utilized to evaluate the enzymes activity afterwards [66].

## Enzymes assay

### Determination of phosphoglucose isomerase (PGI) activity

According to Zhou et al. [47], the PGI standard assay (EC: 5.3.1.9) was carried out. The reaction was assessed by involving glucose-6-phosphate dehydrogenase (G6PDH) in a reaction mixture containing 0.2 ml of 2.5 mM MgCl_2_, 1.5 ml of 50 mM HEPES (pH 7.0), 0.2 ml of 1 mM EDTA, 0.2 ml of 0.5 mM NADP, 0.5 ml of 1.5 mM F6P, 0.4 ml of enzyme extract, and 0.5 units of G6PDH, in a 3 ml final volume. The mixture was incubated for 5 min at 30 °C. At 340 nm, NADPH formation was detected. Under the experimental conditions, one unit of enzyme is equated to 1 μM NADPH reduced per minute.

### Determination of pyruvate dehydrogenase (PDH) activity

According to Gohil and Jones [67], the PDH (EC. 1.2.4.1) assay mixture contained the following components in 3 ml volume: 1.5 ml of 100 mM Tris-HCl (pH 7.0), 0.2 ml of 0.5 mM EDTA, 0.2 ml of l mM MgSO_4_, 0.2 ml of 0.5 mM NAD, 0.2 ml of 0.5 mM CoASH, 0.2 ml of 1 mM thiamin pyrophosphate, 0.2 ml of 1 mM pyruvate, and 0.3 ml of enzyme extract. At 340 nm, the absorbance was then measured.

### Determination of glucose-6-phosphate dehydrogenase (G6PD) activity

The activity of G6PD (EC 1.1.1.49) was measured by Betke et al. [68] approach which is dependent on the spectrophotometric measurement of NADPH formation at 340 nm. The reaction mixture was in 3 ml volume, which contained 1.5 ml of 10 mM MgCl_2_, 0.5 ml of 0.6 mM G6P, 0.5 ml of 0.2 mM NADP, and 150 mM Tris-HCl buffer (pH 8.0). About 0.5 ml of the enzyme extract was added to begin the reaction. The amount of enzyme needed to reduce 1 μM of NADP per minute is equal to one unit (U) of G6PD activity.

### Determination of nitrate reductase (NR) activity

According to Lewis et al. [69], in a reaction mixture containing 100 mM KH_2_PO_4_ (pH 7.0), 100 mM KNO_3_, and 0.5 ml of enzyme extract, nitrate reductase (NR) (EC.1.7.1.1) activity was evaluated. At 540 nm, the activity was spectrophotometrically measured. Using varied NaNO_2_ concentrations, the calibration curve was performed.

### Determination of total soluble protein content

The Bradford method [70] was utilized to determine the amount of total soluble protein. In a tube containing 1 ml of Bradford’s reagent, 30 μl of each treated extract was added and thoroughly mixed. The absorbance was then measured at 595 nm. Using the bovine serum albumin (BSA) calibration curve as a standard, the protein concentration in the sample was determined.

### Cytotoxic activity

The cytotoxic potency of the biosynthesized SeNPs and Se/Ch-nanoconjugate was evaluated using HepG2 (Hepatocellular carcinoma cell line), Caki-1 (HTB-46) (Renal cell carcinoma cell line), and WI-38 (Lung fibroblast cell lines). The 3-(4,5-dimethylthiazol-2-yl)-2,5-diphenyl tetrazolium bromide (MTT) colorimetric assay was used to assess the cell lines viability [71]. It depends on the yellow color change of the MTT solution into blue insoluble formazan crystals by mitochondrial succinate dehydrogenase. It has been demonstrated that the viable cells number is directly proportional with the MTT color reduction of the proper blue formazan [72].

The cell lines were cultured in RPMI-1640 media with 10% fetal bovine serum. Then, 100 μg/ml of streptomycin and 100 μg/ml of penicillin were added. The cell lines were inoculated in a 96-well plate with 1.0 × 10^4^ cells per well. The inoculated plates were incubated for 48 h with 5% CO_2_ at 37 °C. After incubation, the cells were treated by various concentrations of the tested NPs and incubated for another 24 h. The MTT solution (5 mg/ml) was then added. The generated blue formazan was dissolved by adding 100 μl of DMSO to each well. The absorbance was measured at 570 nm, and the colorimetric analysis was obtainable by EXL 800, USA. Data were given as a mean ± SD of three independent experiments.

The % inhibitions were calculated as following:

$$\textrm{Percentage}\ \textrm{viability}=\left(\textrm{At}-\textrm{Ab}\right)\times 100/\left(\textrm{Ac}-\textrm{Ab}\right)$$

$$\textrm{Percentage}\ \textrm{inhibition}=\left(100-\textrm{At}-\textrm{Ab}\right)\times 100/\left(\textrm{Ac}-\textrm{Ab}\right)$$

Where, At_=_ the absorbance value of tested NPs, Ab_=_ the absorbance value of blank, and Ac_=_ the absorbance value of negative control (untreated cells).

The negative control was prepared by adding untreated cells to MTT solution and solubilizing buffer, whereas the blank sample was obtained by mixing a medium free of cells with solubilizing buffer and MTT solution.

## Statistical analysis

The SPSS V-20 software was used to compute and compare the means and SDs (standard deviations) of all experiments, which were all run in triplicate. One-way ANOVA was used to determine the differences significance at *p* ≤ 0.05. The IC_50_ values were obtained from a sigmoid-type nonlinear regression that was processed via the GraphPad 8.2.4® program.

## Results

### Biosynthesis of SeNPs and UV–visible spectroscopic analysis

**Fig. 1:**
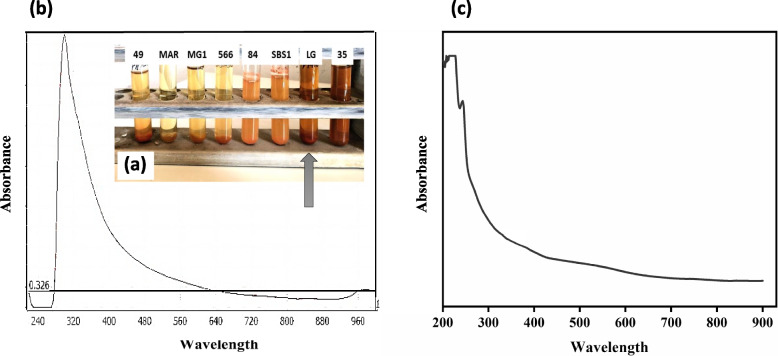
**a** The visual appearance of eight actinomycetes supernatants containing Na_2_SeO_4_ after 48 h of incubation. UV–Visible absorption spectra of **b** biosynthesized SeNPs, and **c** supernatant (LG)

In the current study, eight marine actinomycetes strains, coded as LG, MAR, 35, SBS1, 84, 49, 566, and MG1, were screened for their ability to extracellularly synthesize SeNPs. After Na_2_SeO_4_ had been added, there were some supernatants that changed their color from pale yellow to deep orange within 48 h of incubation, providing preliminary evidence of SeNPs synthesis (Fig. 1a). It was also observed that the supernatants orange colors varied in their intensities. Based on the color changing, one isolate actinomycete coded as LG was selected as a potential candidate for SeNPs production. For further confirmation, the UV-visible spectra analysis was conducted. The biosynthesized SeNPs UV-Visible spectrum showed a characteristic strong plasmon peak at 300 nm compared to the supernatant (LG) spectrum (Fig. 1b, c).

### Identification of potential isolate

**Fig. 2:**
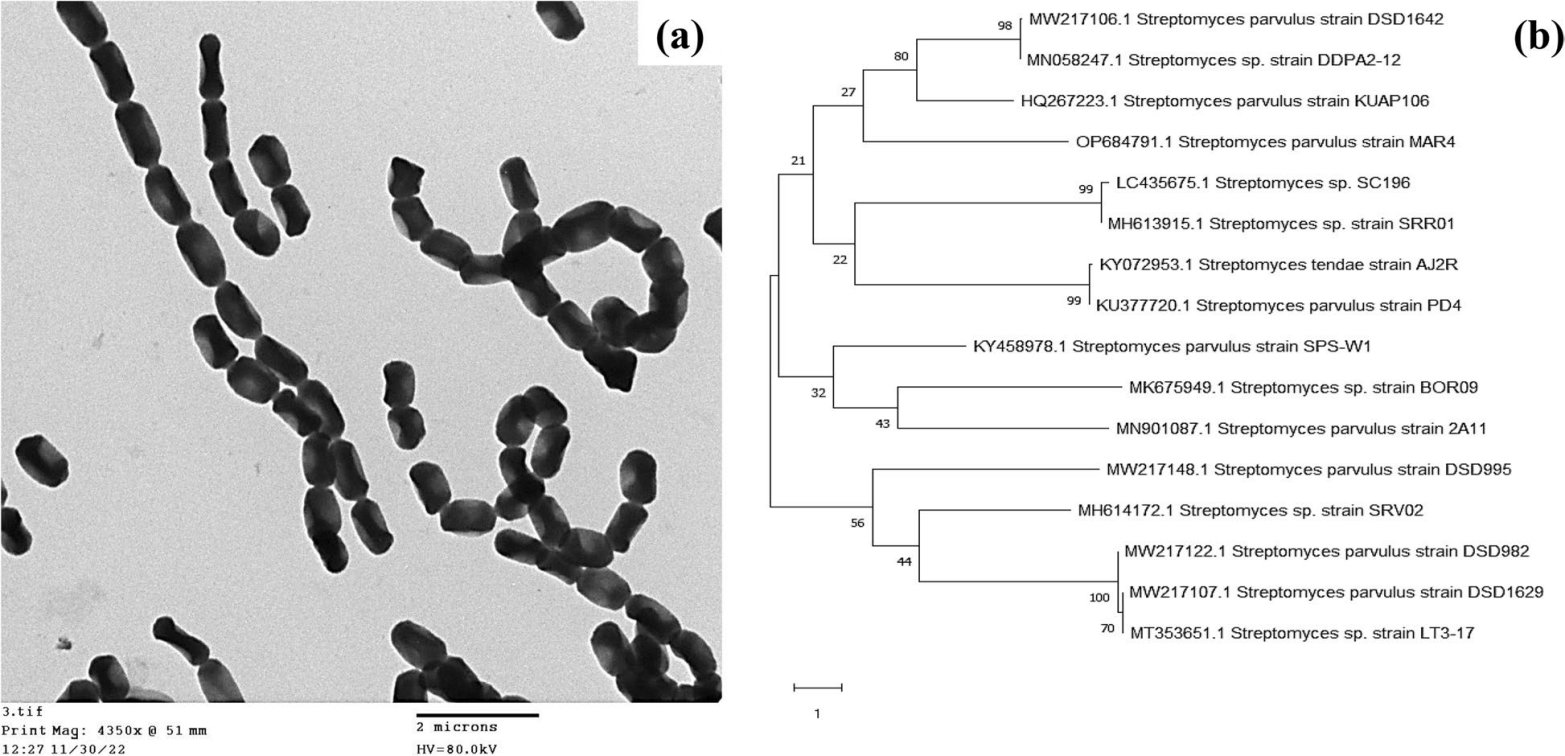
*Streptomyces parvulus* MAR4 identification; **a** Its spore chain morphology TEM micrograph and **b** Constructed phylogenetic tree

The LG actinomycete isolate was identified morphologically using TEM imaging. The TEM micrograph showed a rectus and aerial flexible substrate mycelium with spiral spore chain and smooth spore surface (Fig. 2a). The LG isolate was classified as a member of *streptomyces* genus. For confirmation, molecular genetic identification was performed. The 16S rRNA was extracted, sequenced, and blasted with sequences already present in GenBank. The sequencing data revealed that the LG isolate shared a 99% similarity with *Streptomyces parvulus*. The nucleotide sequence was deposited into GenBank under the name *Streptomyces parvulus* MAR4 with accession number OP684791. Using MEGA-X software, a phylogenetic tree was constructed by the blast result as well as adjacent genus and species (Fig. 2b). According to the phylogenetic analysis, LG isolate and *Streptomyces parvulus* are closely linked genetically.

### XRD analysis

**Fig. 3:**
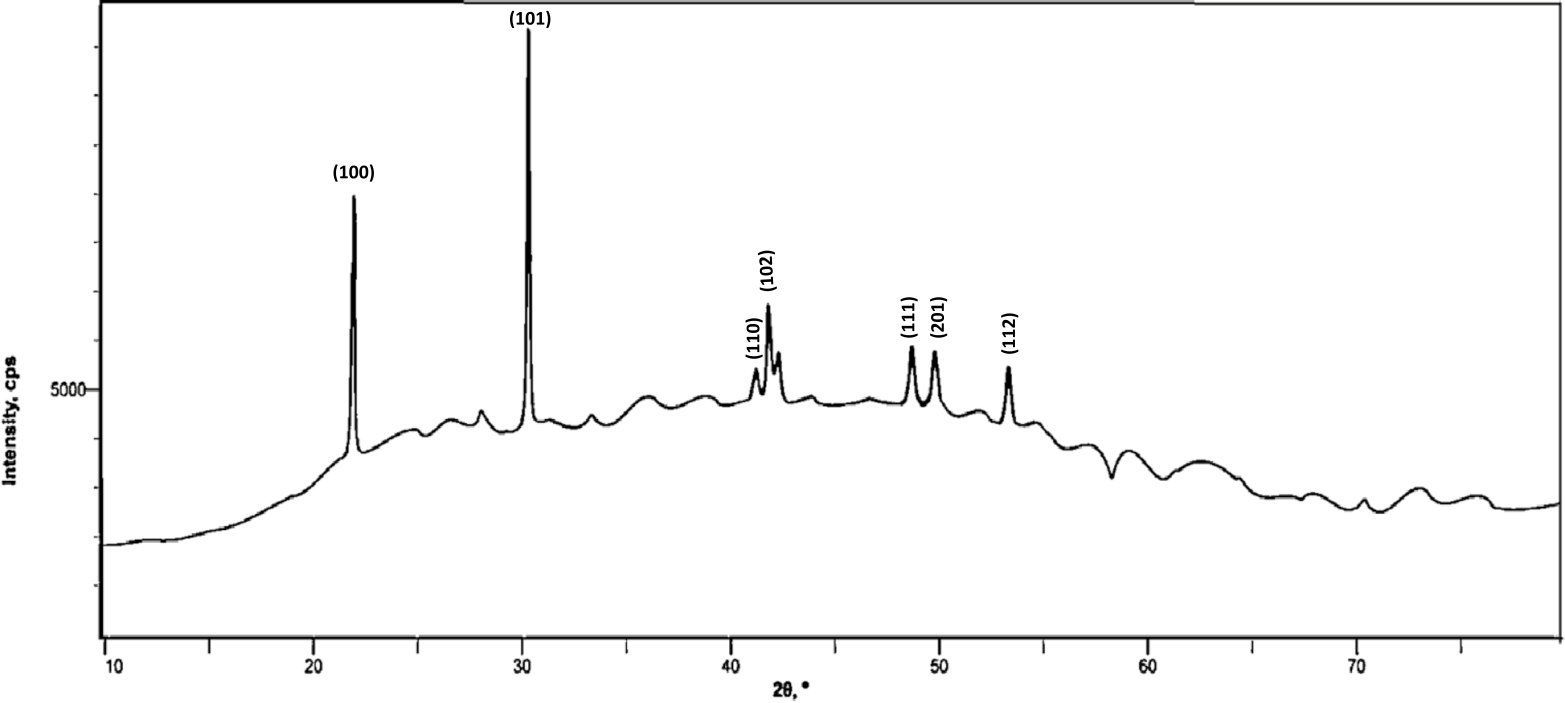
XRD diffractogram of SeNPs extracellularly synthesized by *Streptomyces parvulus* MAR4

Using the XRD technique, the crystal structure of the biosynthesized SeNPs was identified, as shown in Fig. 3. The diffraction peaks at 2θ values of 22.84° (100), 30.25° (101), 41.15° (110), 42.22° (102), 48.24° (111), 49.72° (201), and 53.21° (112) were perfectly indexed to crystalline selenium. The crystalline nature of the biosynthesized SeNPs was verified by the joint committee on powder diffraction standards (JCPDS) file number 06–0362.

### FTIR analysis

**Fig. 4:**
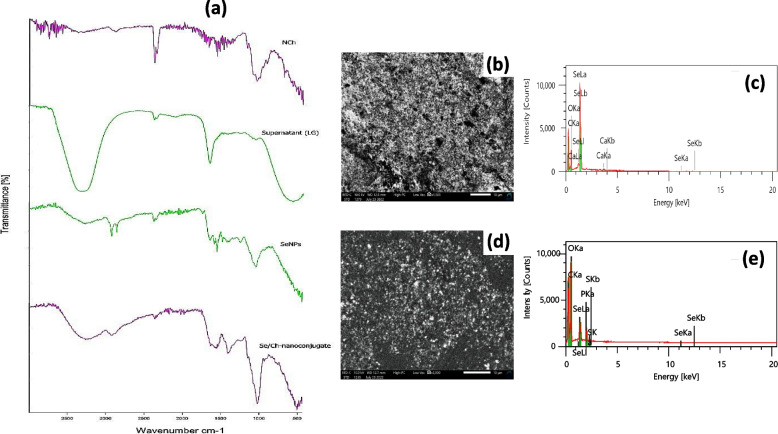
**a** FTIR spectra of synthesized nanoparticles; nano-chitosan (NCh), supernatant of *Streptomyces parvulus* MAR4 (supernatant (LG)), biosynthesized selenium nanoparticles by *Streptomyces parvulus* MAR4 supernatant (SeNPs), and their nanoconjugate (Se/Ch-nanoconjugate). SEM-EDX analyses for biosynthesized SeNPs (**b** and **c**, respectively) and Se/Ch-nanoconjugate (**d** and **e**, respectively)

The function groups were identified using Fourier transform infrared spectroscopy (FTIR) which also provided crucial information about the chemical interactions of *Streptomyces parvulus* MAR4 supernatant (supernatant (LG)), biosynthesized SeNPs, NCh, Se/Ch-nanoconjugate. In the NCh spectrum, the distinct peaks at 3447, 2826, 1643, 1335, and 1150 cm^−1^ were observed and attributed to O–H stretching, C–H stretching vibration, N–H bending, C–N stretching, and C–O–C stretching vibration, respectively (Fig. 4a, NCh).

The functional groups necessary for the Se^+^ reduction to SeNPs were detected through the supernatant (LG) FTIR spectrum (Fig. 4a, supernatant (LG)). Its designative peaks were found at 3320, 2361, 1634, 1090, and 570 cm^−1^, which were respectively corresponded to N–H and O–H stretching, O=C=O bending, C–N and C–C stretching, C–O stretching, and haloalkanes.

Figure 4a, SeNPs depicts the FTIR spectrum of biosynthesized SeNPs. After interaction with SeNPs, many distinct peaks in the supernatant (LG) spectrum were moved, vanished, or varied in intensities, indicating SeNPs production. Additionally, new bands appeared at 2580, 2850, and 1000:1625 cm^−1^, confirming the formation of new bonds between supernatant (LG) biomolecules and SeNPs.

There were several distinctive peaks from each individual agent (supernatant (LG), NCh, and SeNPs) in the FTIR spectrum of Se/Ch-nanoconjugate, demonstrating their strong interactions (Fig. 4a, Se/Ch-nanoconjugate). Furthermore, many peaks in Se/Ch-nanoconjugate spectrum were merged, shifted, or altered from their parental ones.

### TEM analysis

**Fig. 5:**
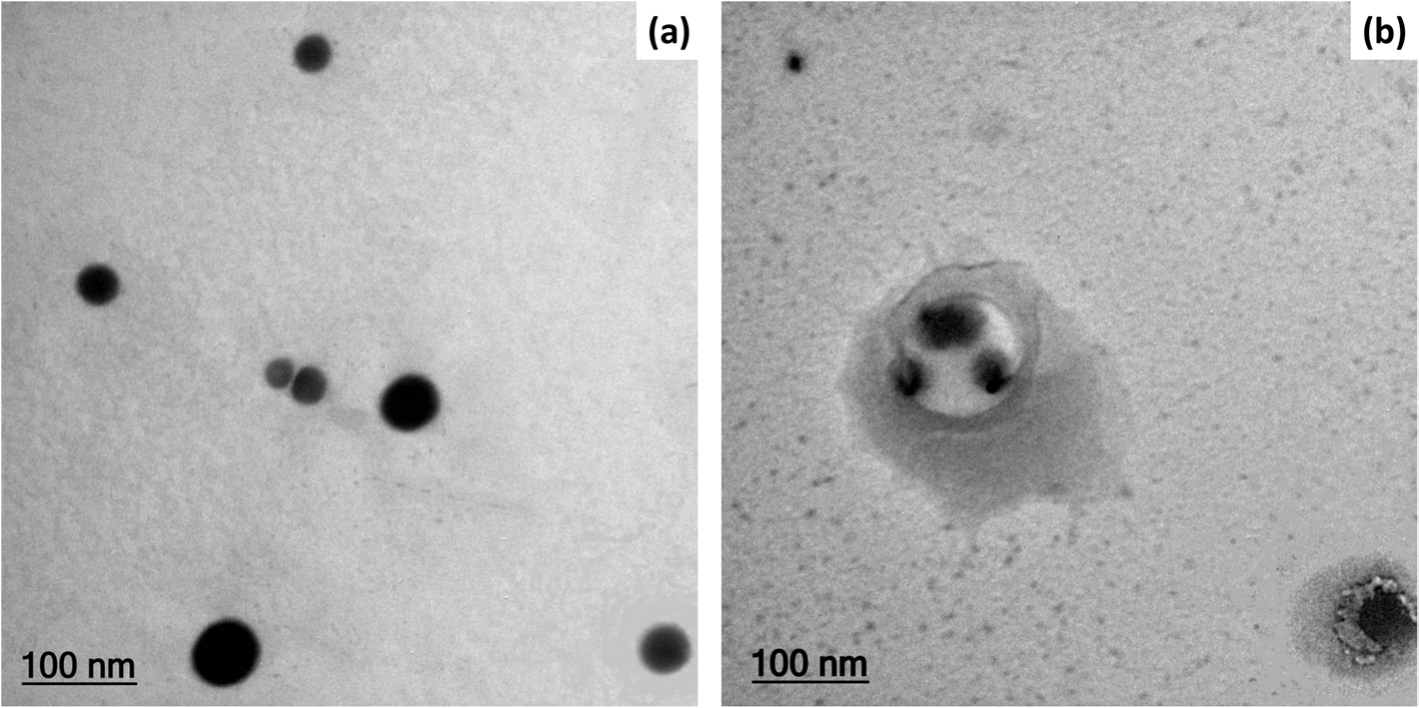
TEM micrographs of **a** biosynthesized SeNPs and **b** Se/Ch-nanoconjugate

The biosynthesized SeNPs and Se/Ch-nanoconjugate TEM micrographs are exhibited in Fig. 5a, b. The SeNPs TEM micrograph revealed their spherical shape, homogeneous distribution, and lacking aggregation (Fig. 5a). Moreover, their diameter was found to be between 48.8 and 129 nm with an average of approximately 94.2 nm. However, the TEM micrograph for Se/Ch-nanoconjugate demonstrated its semispherical shape and well distribution; its diameter ranged from 39.7 to 98.1 nm with an average of 74.9 nm (Fig. 5b).

### SEM and EDX analyses

The SEM-EDX analyses for SeNPs and Se/Ch-nanoconjugate were performed. The SEM micrograph of SeNPs clearly demonstrated their small and spherical shape (Fig. 4b). Furthermore, their EDX analysis revealed the presence of peaks for selenium (50.41%), calcium (2.34%), oxygen (15.78%), and carbon (31.29%), which were likely contributed by supernatant (LG) (Fig. 4c). Selenium was proved to be the predominant element in the sample.

Figure 4d shows the SEM micrograph of Se/Ch-nanoconjugate which confirmed its approximately spherical shape and nanometric scale. The nanoconjugate SEM micrograph showed less agglomerations than those presented in the SeNPs. The presence of selenium (11.87%), phosphorus (7.08%), carbon (32.54%), sulfur (1.90%), and oxygen (46.62%) elements were established by the given nanoconjugate EDX diagram (Fig. 4e). Consequently, there was an agreement between the TEM and SEM results for both samples.

### Hydrodynamic diameter and zeta (ζ) potential

The hydrodynamic diameter, PDI, and ζ potential of SeNPs and Se/Ch-nanoconjugate are depicted in Table 1. The mean hydrodynamic diameter of SeNPs was found to be 196,2 ± 38 nm while Se/Ch-nanoconjugate mean hydrodynamic diameter was larger (476.6 ± 245 nm), indicating successful conjugation and integrations.

**Table 1:** Hydrodynamic diameter, polydispersity index (PDI), and zeta (ζ) potential of biosynthesized SeNPs and Se/Ch-nanoconjugate

NPs	Mean hydrodynamic diameter (nm)	PDI	ζ potential (mV)
**SeNPs**	196,2	0.252	37.08
**Se/Ch-nanoconjugate**	476.6	0.264	55.91

The solution homogeneity is estimated by the polydispersity index (PDI). PDI value ranges from 0 to 1, which 0 represents an ideal solution with same-sized particles, and 1 represents a highly polydisperse solution with various sized particles. Values below 0.5 are regarded as monodispersed, whereas those above 0.7 are regarded as substantially polydisperse [73]. SeNPs and Se/Ch-nanoconjugate showed PDI values below 0.5 (0.252 and 0.264, respectively), indicating that they were monodispersed, stable, and uniformly sized in the solution.

By measuring the ζ potential of the colloidal solution, the electric potential surrounding the particle, the stability was assessed. The solution is regarded as stable if the ζ potential is greater than ±30 mV, and below this value, is considered as unstable and more likely to flocculate. The ζ potential of SeNPs was found to be 37.08 mV, which is stable, while Se/Ch-nanoconjugate ζ potential was 55.91, which is much more stable.

### Antimicrobial activity

**Fig. 6:**
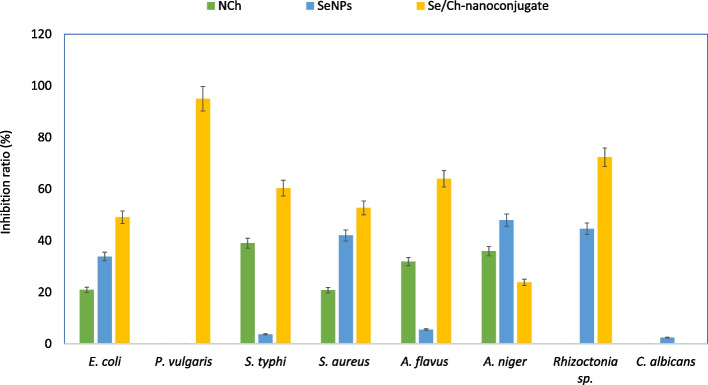
Antimicrobial activity of synthesized nanoparticles against wide range of microbial pathogens

The biosynthesized SeNPs, NCh and Se/Ch-nanoconjugate were evaluated for their antibacterial and antifungal activity against different microbial pathogens (*Escherichia coli*, *Proteus vulgaris*, *Salmonella typhi*, *Staphylococcus aureus*, *Aspergillus flavus*, *Aspergillus niger*, *Rhizoctonia* sp., and *Candida albicans*). The results obtained demonstrated that the synthesized NPs have a broad spectrum of antimicrobial activity against both bacterial and fungal strains (Fig. 6). The Se/Ch-nanoconjugate showed the highest effectiveness and exhibited superior antimicrobial activity against most tested microbial pathogens. The Se/Ch-nanoconjugate showed stronger antibacterial than antifungal action.

The nanoconjugate exhibited strong antimicrobial activity against *S. typhi*, *P. vulgaris, E. coli*, *S. aureus*, *A. flavus*, and *Rhizoctonia* sp.; *P. vulgaris* recorded the highest sensitivity, but in contrast, it presented the lowest one toward both NCh and SeNPs. However, *C. albicans* was the most resistant against all tested agents. The SeNPs and NCh exhibited moderate to strong antimicrobial activity, but SeNPs were more powerful. As a result, the order of tested agents according to their antimicrobial activities can be considered as following: NCh < SeNPs< Se/Ch-nanoconjugate.

### Influence of synthesized NPs on metabolic enzymes activity of tested microbial pathogens

The biosynthesized SeNPs, NCh and Se/Ch-nanoconjugate expressed various inhibitory effects against four metabolic key enzymes (PGI, PDH, G6PDH, and NR) of the tested microbial pathogens, and the Se/Ch-nanoconjugate was the most suppressive (Tables 2, 3, 4 and 5). The four tested enzymes in *A. flavus, Rhizoctonia* sp., *E. coli*, *P. vulgaris*, *S. typhi*, and *S. aureus* were incredibly inhibited by the nanoconjugate, except those present in *Candida albicans* which showed high resistance toward all tested NPs. On the other hand, the tested enzymes were moderately to strongly inhibited by SeNPs and NCh; nevertheless, the NCh showed less inhibitory effect than SeNPs. Obviously, the synthesized NPs order according to their inhibitory effects against the tested enzymes can be considered as following: NCh < SeNPs < Se/Ch-nanoconjugate. Therefore, there is an agreement with the data obtained by antimicrobial test.

**Table 2:** Effect of synthesized NPs on phosphoglucose isomerase (PGI) activity in a wide range of microbial pathogens

Tested NPs	% Inhibition of PGI							
	*E. coli*	*P. vulgaris*	*S. typhi*	*S. aureus*	*A. flavus*	*A. niger*	*Rhizoctonia* sp.	*C. albicans*
**NCh**	30.0 ± 0.7	0.6 ± 0.01	35.1 ± 0.8	21.9 ± 0.7	31.7 ± 0.7	30.7 ± 0.7	6.8 ± 0.3	11.3 ± 0.4
**SeNPs**	30.9 ± 0.7	2.2 ± 0.1	5.0 ± 0.2	44.0 ± 0.8	6.7 ± 0.2	45.8 ± 0.8	48.9 ± 0.8	2.1 ± 0.2
**Se/Ch-nanoconjugate**	43.8 ± 0.9	82.4 ± 1.5	60.0 ± 1.2	53.3 ± 1.0	61.3 ± 1.3	26.1 ± 0.5	71.6 ± 1.4	1.4 ± 0.01

**Table 3:** Effect of synthesized NPs on pyruvate dehydrogenase (PDH) activity in a wide range of microbial pathogens

Tested NPs	% Inhibition of PDH							
	*E. coli*	*P. vulgaris*	*S. typhi*	*S. aureus*	*A. flavus*	*A. niger*	*Rhizoctonia* sp.	*C. albicans*
**NCh**	23.9 ± 0.7	1.5 ± 0.01	39.0 ± 0.8	21.9 ± 0.6	29.1 ± 0.5	35.0 ± 0.7	8.3 ± 0.3	6.3 ± 0.3
**SeNPs**	40.7 ± 0.8	1.0 ± 0.01	6.5 ± 0.2	46.5 ± 0.8	5.5 ± 0.2	48.6 ± 0.6	51.5 ± 1.3	5.0 ± 0.2
**Se/Ch-nanoconjugate**	46.2 ± 0.9	75.0 ± 1.5	66.7 ± 1.2	50.0 ± 1.0	59.4 ± 0.9	29.9 ± 0.6	75.7 ± 1.3	1.3 ± 0.01

**Table 4:** Effect of synthesized NPs on glucose 6-phosphate dehydrogenase (G6PDH) activity in a wide range of microbial pathogens

Tested NPs	% Inhibition of G6PDH							
	*E. coli*	*P. vulgaris*	*S. typhi*	*S. aureus*	*A. flavus*	*A. niger*	*Rhizoctonia* sp.	*C. albicans*
**NCh**	31.6 ± 0.7	1.2 ± 0.01	35.9 ± 0.7	23.4 ± 0.5	27.2 ± 0.6	37.1 ± 0.7	16.9 ± 0.4	10.4 ± 0.3
**SeNPs**	35.0 ± 0.7	2.0 ± 0.01	6.6 ± 0.2	38.9 ± 0.9	5.8 ± 0.2	51.6 ± 0.9	66.1 ± 1.5	3.1 ± 0.2
**Se/Ch-nanoconjugate**	40.0 ± 0.8	86.7 ± 1.6	55.0 ± 1.0	47.0 ± 0.8	66.0 ± 1.2	24.7 ± 0.6	57.6 ± 1.0	4.5 ± 0.2

**Table 5:** Effect of synthesized NPs on nitrate reductase (NR) activity in a wide range of microbial pathogens

Tested NPs	% Inhibition of NR							
	*E. coli*	*P. vulgaris*	*S. typhi*	*S. aureus*	*A. flavus*	*A. niger*	*Rhizoctonia* sp.	*C. albicans*
**NCh**	30.6 ± 0.6	0.9 ± 0.1	28.2 ± 0.5	24.8 ± 0.6	23.1 ± 0.6	30.7 ± 0.6	13.6 ± 0.4	8.0 ± 0.4
**SeNPs**	35.0 ± 0.7	2.0 ± 0.01	3.4 ± 0.1	42.0 ± 0.8	5.1 ± 0.2	40.0 ± 0.8	57.7 ± 1.0	2.1 ± 0.2
**Se/Ch-nanoconjugate**	50.0 ± 1.3	90.0 ± 1.8	60.0 ± 1.4	53.6 ± 0.9	65.8 ± 1.3	31.6 ± 0.7	67.1 ± 1.4	4.9 ± 0.2

### Cytotoxic activity

**Fig. 7:**
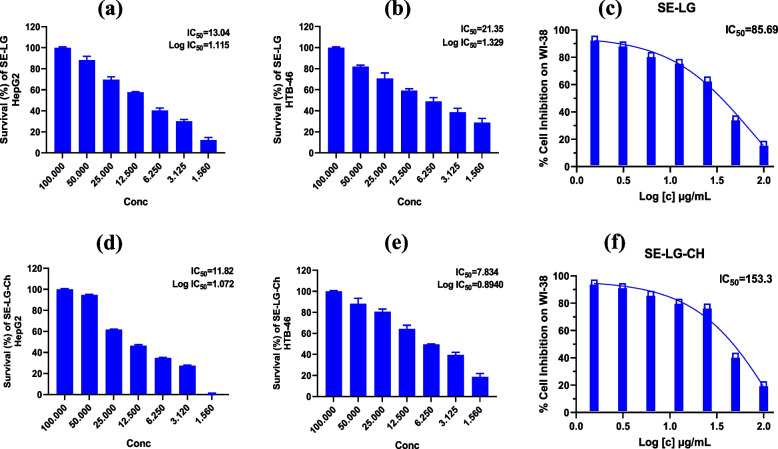
IC_50_ of synthesized nanoparticles; IC_50_ of SeNPs biosynthesized by *Streptomyces parvulus* MAR4 supernatant against cell viability of HepG2 (Hepatocellular carcinoma cell line), Caki-1 (HTB-46) (Renal cell carcinoma cell line), and WI-38 (Lung fibroblast cell lines) (**a**, **b**, and **c**, respectively). IC_50_ of Se/Ch-nanoconjugate against the same three cell lines (**d**, **e**, and **f**, respectively). Each value represents the mean ± SD of three independent experiments

In this study, we used an in vitro MTT colorimetric test to determine the cytotoxic potential of the biosynthesized SeNPs and Se/Ch-nanoconjugate. The cytotoxic activity was evaluated against HepG2 and Caki-1 (HTB-46) tumor cell lines and WI-38 normal cell lines (Table 6, Fig. 7). Precisely, as shown in Table 6, SeNPs demonstrated significant cytotoxicity against HepG2 (IC_50_ = 13.04 μg/ml), moderate toxicity against Caki-1 (HTB-46) (IC_50_ = 21.35 μg/ml), and low cytotoxicity against WI-38 (IC_50_ = 85.69 μg/ml). However, the Se/Ch-nanoconjugate exhibited substantial cytotoxicity against HepG2 and Caki-1 (HTB-46) with corresponding IC_50_ values of 11.82 and 7.83 μg/ml, respectively. However, it exhibited very low cytotoxicity on WI-38 with IC_50_ of 153.3 μg/ml, indicating its safety and selectivity. Consequently, Se/Ch-nanoconjugate was demonstrated to have efficient and superior anticancer activity.

**Table 6:** Cytotoxic effects of biosynthesized SeNPs by *Streptomyces parvulus* MAR4 supernatant and Se/Ch-nanoconjugate against HePG-2 and Caki-1 (HTB-46) cell lines

Tested NPs	HePG-2	In vitro Cytotoxicity, IC_50_ (μg/ ml) ^[a]^	
		HTB-46	WI-38
**SeNPs**	13.04	21.35	85.69
**Se/Ch-nanoconjugate**	11.82	7.83	153.3

^[a]^IC_50_ (μg): 1–5 (very strong); 6–15 (strong); 16–50 (moderate); 51–100 (weak), and > 100 (non-cytotoxic). Abbreviations: HepG2: Hepatocellular carcinoma cell line; Caki-1 (HTB-46): Renal cell carcinoma cell line; WI-38: Lung fibroblast cell lines

## Discussion

Actinomycetes are considered to be good producers for active molecules which serve as a reducing and stabilizing agent in NPs synthesis [16]. Accordingly, eight isolated actinomycetes were screened to synthesize SeNPs extracellularly. Some supernatants color changed from pale yellow to deep orange as a first indication of SeNPs synthesis [54, 55]. Moreover, the supernatants varied in their orange colors intensity, reflecting that each actinomycete produced its own unique SeNPs size [74]. The presence of surface plasmon resonance (SPR) in the biosynthesized SeNPs was responsible for the distinctive deep orange color of the reaction mixtures [75]. The UV-Visible spectral comparison between the supernatant (LG) and biosynthesized SeNPs spectrum clearly demonstrated the NPs formation. The UV-Visible spectrum of the biosynthesized SeNPs showed a sharp absorption peak at 300 nm which is consistent with previous studies for bacterially-synthesized SeNPs [76, 77]. Conversely, the UV–visible spectra of SeNPs extracellularly synthesized by *Pseudomonas aeruginosa* and *Bacillus cereus* exhibited different absorption peaks at 520 nm and 590 nm, respectively [78, 79]. Consequently, UV-visible spectra of biosynthesized SeNPs vary depending on their atomic structure [54].

The XRD profile of the biosynthesized SeNPs established the crystalline structures of SeNPs [80–82]. The amorphous structures, without diffraction peaks, possibly related to the formation of amorphous and monocyclic SeNPs [82, 83]. The sharpening of the peaks obviously revealed that the particles were spherical in shape [54].

The most effective bonds and groups in the synthesized NPs were detected using FTIR analysis. For the NCh spectrum, the main characteristic bands of the natural chitosan were observed [84–86]. The most noticeable bands in Fig. 4a, NCh were specific to NCh biochemical bonding in earlier studies [87, 88]. Additionally, the P=O stretching at 1196 cm^−1^ indicated the subsequent NCh synthesis after TPP interaction [57].

The supernatant (LG) FTIR spectrum confirmed the presence of biomolecules responsible for Se^+^ reduction and subsequent SeNPs synthesis (Fig. 4a, supernatant (LG)). NADH-dependent enzymes and/or Sulfur-containing proteins, which had superb redox characteristics serving as cofactors in selenide ions reduction, were exemplified by OH and NH stretching at 3320 cm^−1^ [79, 89]. The presence of proteins was indicated by C–N and C–C stretching at 1634 cm^−1^ [36]. Additionally, the Protein carbonyl stretching was demonstrated by the bands between 1090 cm^−1^ and 1020 cm^−1^ [79]. Subsequently, the supernatant (LG) proteins could act as the main reducing or capping agents in SeNPs synthesis [90].

The biosynthesis of SeNPs and formation of novel bonds with supernatant (LG) biomolecules were revealed by the SeNPs FTIR spectrum (Fig. 4a, SeNPs). The broad strong band at 3320 cm^−1^ in the supernatant (LG) spectrum became very weak in SeNPs spectrum, demonstrating reduction of selenide ions producing SeNPs [45]. The supernatant (LG) proteins involved in the production of SeNPs were evidenced by the band shifting from 1634 cm^−1^ to 1650 cm^−1^ and changing in intensity. Moreover, the detection of numerous notable new bands at 2580, 2850, and around 1500 cm^−1^ demonstrated the presence of strong new interactions between supernatant (LG) biomolecules and SeNPs [57, 91].

The Se/Ch-nanoconjugate FTIR spectrum revealed identical peaks from each individual agent (Fig. 4a, Se/Ch-nanoconjugate). However, other peaks were moved, merged, or changed from their origins. Therefore, both chemical and physical interactions during the conjugation of NCh with SeNPs could be clearly shown by the spectral comparison [92].

The structural features of SeNPs and Se/Ch-nanoconjugate were screened via TEM analysis. For SeNPs, the TEM micrograph confirmed their well distribution and spherical shape with 94.2 nm mean diameter (Fig. 5a). In recent studies, the biosynthesized SeNPs diameters were found to be between 45 to 250 nm [55, 93, 94]. However, Se/Ch-nanoconjugate had a semispherical shape with a mean diameter of 74.9 nm (Fig. 5b), indicating NCh capacity to act as a capping agent [95].

The SEM–EDX analyses were performed for SeNPs and Se/Ch-nanoconjugate to further investigate their morphology and elemental content. The SEM–EDX analyses for SeNPs demonstrated their spherical shape and prevalence of selenium element (Fig. 4b, c), which are in accordance with SeNPs biosynthesized by *Trichoderma atroviride*, *Mariannaea* sp., *Synechococcus leopoliensis*, and *Anabaena* sp. PCC 7120 [93, 94, 96, 97]. Nevertheless, the Se/Ch-nanoconjugate showed less agglomeration in SEM micrographs, indicating the aggregation and well distribution of SeNPs into the polymeric matrix (Fig. 4d). The presence of phosphorus in the nanoconjugate EDX diagram (Fig. 4e) confirmed the formation of NCh by cross-linking with TPP [98, 99].

Both TEM and DLS size distributions show a clear inconsistency. This could be explained by following: the hydrodynamic diameter, measured by DLS, is representative for the size of a hypothetical sphere that diffuses at the same rate as the particles being examined, while the diameter measured via TEM really provided the physical size of the particle. According to the Rayleigh approximation, the scattered intensity is proportional to d^6^ (particle diameter). Consequently, the light scattered by the largest particles could hide the light scattered by the smallest ones. For example, consider two particles, one of which has a diameter ten times greater than the other. According to the d^6^ factor, the larger particle will scatter 10^6^ more light than the smaller one [100]. Additionally, the biosynthesized SeNPs and Se/Ch-nanoconjugate showed high ζ potential, reflecting their high stability and dispersity triggered due to electrostatic repulsion between particles [11, 101]. The nanoconjugate demonstrated higher ζ potential, indicating the effectiveness of NCh in capping SeNPs rather than biochemically interacting with them [102].

The NCh FTIR spectrum and nanoconjugate TEM, SEM, and DLS analyses demonstrated that the NCT was successfully accomplished by ionic gelation with TPP crosslinking. This approach generates reversible ionic cross-linking rather than chemical cross-linking through electrostatic interactions between the polymer and ionic gelatinizer, ensuring reduction of toxicological effects on the produced NPs [103]. The NCh produced by this method was demonstrated to have superb properties because of its applications as a bioactive agent or nanocarrier for bioactive compounds [45, 57].

NPs stand out as an exceptionally promising approach in combating MDR pathogens [104]. Herein, the synthesized NPs exhibited potent antimicrobial activity against the most tested microbial pathogens; Se/Ch-nanoconjugate was the most forceful agent, and that was related to the microbial pathogens disability to develop resistance toward multiple combined antimicrobial compounds with various action modes [105, 106]. The powerful antimicrobial activity of the synthesized NPs was further investigated by their inhibitory effect against the four tested metabolic enzymes. Se/Ch-nanoconjugate was proved to be the most suppressive agent. They can interfere with these enzymes, disrupt essential cellular processes, and ultimately lead to the inhibition of microbial growth and proliferation. Therefore, one of the antimicrobial mechanisms of the synthesized NPs appears to be the inhibition of metabolic key enzymes. The *C. albicans* resistance was in accordance with the results obtained by Hashem and Salem [107] and Cremonini et al. [108]. It was also reported that SeNPs had more antifungal activity against filamentous fungi than unicellular fungi [107].

The biosynthesized SeNPs depicted superior antimicrobial activity when compared to their chemically synthesized counterparts [108]. In previous studies, the biosynthesized SeNPs were demonstrated to exhibit potent antimicrobial activity against various microbial pathogens [33, 97, 109, 110]. Four potential mechanisms were proposed by Huang et al. [111]: (1) metabolic invasion through disruption the intracellular adenosine triphosphate (ATP) levels, (2) depolarization, (3) intracellular reactive oxygen species (ROS) values variation, and (4) disturbance of biological membranes. Additionally, SeNPs were proved to have a strong inhibitory effect on α-amylase, α-glucosidase, galactosidase, and protease [112, 113]. Consequently, SeNPs antimicrobial mechanism may involve the deterioration of DNA structure and disruption of enzyme functionality, resulting from the generation of hydroxyl free radicals [114].

The antibacterial action of NCh was reported against various microbial pathogens, and several hypothesized mechanisms were documented [115–117]. The electrostatic interaction between the NCT positively charged amino group and the negatively charged microbial cell membranes is considered to be one of the main antimicrobial mechanisms, which causes cell damage through the leakage of proteinaceous and other intracellular components [118, 119]. The NCh may also act as a chelating agent, binding to trace metals and generating toxins that inhibit microbial growth [120]. Additionally, NCT can carry and control the release of bioactive agents [121].

As a result, the NCh can easily penetrate the microbial cell membrane pores carrying the biosynthesized SeNPs and release them there. Accordingly, the nanoconjugate has direct effects on the cells; it can interfere with ATP synthesis, affect cell division, and cause cell lysis [45, 57, 98, 122]. Furthermore, the substantial positive charge of Se/Ch-nanoconjugate could electrostatically interact with the negatively charged cell membranes causing cell damage. Consequently, the Se/Ch-nanoconjugate was proved to be the most powerful antimicrobial agent.

Recently, biosynthesized SeNPs have been receiving a lot of attention as a potential anticancer agent due to their remarkable biological activity, biocompatibility, and low toxicity [82, 122, 123]. Furthermore, it was reported that they have no side effects on normal cells while specifically inhibiting the tumor growth [124, 125]. In the current study, the NCh was loaded to improve the biological properties of the biosynthesized SeNPs [126]. The biosynthesized SeNPs and Se/Ch-nanoconjugate exhibited outstanding anticancer activity against HepG2 and Caki-1 (HTB-46) tumor cell lines; the nanoconjugate was the most powerful with IC_50_ values of 11.82 and 7.83 μg/ml, respectively, with very low cytotoxicity on normal cells (IC_50_ = 153.3 μg/ml).

SeNPs were proven to have a potential cytotoxic effect against various tumor cell lines [13, 127–129]. Hashem and Salem [107] declared that the biosynthesized SeNPs were shown to inhibit HepG2 with an IC_50_ of 102.8 μg/ml. According to Hassanien et al. [130], the three tumor cell lines; Caco2, HepG2, and Mcf-7, were inhibited by biogenic SeNPs with IC_50_ values of 151, 393, and 252 μg/ml, respectively. Moreover, SeNPs biosynthesized by *Streptomyces griseoruber* were demonstrated to have a cytotoxic effect on HT-29 cell lines [55]. Subsequently, our study demonstrated that the biosynthesized SeNPs and nanoconjugate were more efficacious as anticancer agents compared to others.

Several anticancer mechanisms for SeNPs were proposed by many studies, including mitochondrial dysfunction, apoptosis induction by triggering the caspase or apoptotic proteins, disruption of cellular homeostasis, cell cycle arrest, ROS excessive production, DNA fragmentation, or a combination of these processes [13, 54, 107, 131, 132]. On the other hand, NCh can directly affect tumor cells through inducing apoptosis, reducing cell growth, or disrupting metabolism [133]. Additionally, the positive charge of NCh can balance the negative charge on the tumor cell surface, leading to selective absorption [134]. NCh was also demonstrated to cause HCC cell death in vitro by triggering membrane disruption, reducing negative surface charge, decreasing mitochondrial membrane potential, inducing lipid peroxidation, disturbance the fatty acid layer of the membrane, and causing DNA fragmentation [135].

In Se/Ch-nanoconjugate, the NCh decorated the SeNPs surface charge making it more positive resulting in selective cellular uptake and enhancing the cell membrane-permeating abilities and apoptosis-inducing activities [136–138]. Furthermore, multiple combined bioactive agents can exhibit various action modes, which work synergistically to exert their anticancer effects. Therefore, the nanoconjugate demonstrated a considerable and potent anticancer action with very low cytotoxicity on normal cells.

## Conclusion

The utilization of *Streptomyces parvulus* MAR4 supernatant for SeNPs biosynthesis was found to be a highly efficient, cost-effective, and safe strategy. The conjugation between biosynthesized SeNPs and NCh exhibited remarkable antimicrobial and anticancer activity against the most tested microbial pathogens, and both chosen tumor cell lines without causing any side effects on normal cells. Its antimicrobial and anticancer mechanisms can be attributed to the presence of multiple combined bioactive agents with diverse modes of action. The conjugation also decreased toxicity and enhanced biocompatibility, safety, and biological activity of each individual agent. Furthermore, the nanoconjugate significant positive charge could electrostatically interact with negatively charged cell membranes, resulting in cell damage. Moreover, the inhibition of key metabolic enzymes was proven to be one of their antimicrobial mechanisms through interfering with these enzymes and disrupting metabolic processes, causing inhibition of microbial growth and proliferation. Overall, the Se/Ch-nanoconjugate can be applied as a promising biomedical agent with both antimicrobial and anticancer properties. However, further in-depth studies are required to fully elucidate the precise mechanisms underlying its antimicrobial and anticancer effects.

## Abbreviations

MDR: Multidrug resistant
SeNPs: Selenium nanoparticles
Ch: Chitosan
NCh: nano-chitosan
PGI: Phosphoglucose isomerase
PDH: Pyruvate dehydrogenase
G6PDH: Glucose-6-phosphate dehydrogenase
NR: Nitrate reductase
TPP: tripolyphosphate
TEM: Transmission electron microscopy
SEM: Scanning electron microscopy
EDX: Energy dispersive X-ray
FTIR: Fourier transform infrared spectroscopy
CCG: Carbon-coated copper grids
LB: Lysogenia broth
HepG2: Hepatocellular carcinoma cell line
Caki-1: Renal cell carcinoma cell line
ROS: Reactive oxygen species
FBS: Foetal Bovine Serum
